# Supplementary material for: Visualising health risks with medical imaging for changing recipients’ health behaviours and risk factors: Systematic review with meta-analysis
Source: PLoS Med. 2022 Mar 3;19(3):e1003920. doi: 10.1371/journal.pmed.1003920 (PMC8893626; doi:10.1371/journal.pmed.1003920)
Supplement: S1 Text — Review protocol (Appendix A); Database search strategies (Appendix B); Outcome selection process (Appendix C). (DOCX) [file pmed.1003920.s001.docx]

**SUPPORTING INFORMATION**

**Review protocol (Appendix A); Database search strategies (Appendix B); Outcome selection process (Appendix C)**

------------------------------------------------------------------------------------------------------------------------

**Appendix A –**

**Impact of visualising health risk with medical imaging on recipients’ health-related behaviours and outcomes: protocol for a systematic review with meta-analysis**

***NB This Appendix details the review methods only, and does not provide the full background to the review. For the complete protocol documents including this background, please see the OSF Project page (***[***https://osf.io/bsf5e/***](https://osf.io/bsf5e/)***).***

In this review we will assess the impact of feeding back medical imaging results that visualise health risks on risk-reducing behaviours and corollary risk factors and health-related outcomes, updating a Cochrane Review published in 2010 [1]. In essence, interventions of this type consist of an individual being shown medical images of his or her body together with an explanation of what is portrayed, typically emphasising the implications of the results and how any health risks can be reduced by the individual changing his or her behaviour. The review will also assess data on adverse events and potential harms, beyond behavioural or health impacts in the undesired direction, such as excessive or enduring levels of anxiety and depression. Since the initial publication of the Cochrane Review, we are aware that a number of key clinical trials have been conducted, and so updating this systematic review will enable a substantially more robust and precise estimate of the effects of this type of intervention.

**OBJECTIVES**

**Primary objective**

To assess the extent to which communicating to individuals images of their own bodies created during medical imaging procedures increases or decreases risk-modifying health behaviours. This will be considered in comparison to the impact of communicating an individual’s health status in a way which does not involve showing them the source images derived from the imaging procedure (such as providing risk information in a different form (e.g. oral feedback, written report), or providing no risk information at all).

**Secondary objective**

To determine the impact on individuals’ related risk factors and health-related outcomes, and on adverse events and harms.

**METHODS**

**Criteria for considering studies for this review**

***Types of studies***

Randomised controlled trials.

***Types of participants***

Participants are adults (18 years and over) who undergo medical imaging procedures assessing current health status, risk of disease or of an existing condition, for which personal risk may be reduced by modification of current behaviour. Women receiving prenatal scanning will be excluded.

***Types of interventions***

The sole or principal component of the intervention is the feedback of an individual’s medical imaging results. Medical imaging is defined in relation to the MeSH definition of diagnostic imaging, but applied without the consideration of diagnostic intent: “Any visual display of structural or functional patterns of organs or tissues (for diagnostic evaluation)” [2]. The specific procedures encompassed under this definition, in line with MeSH organisation, include magnetic resonance imaging (MRI), computed tomography (CT), radiography, ultrasonography, and ultraviolet (UV) photography. We will determine whether medical imaging feedback is the principal component by reference to the described intervention components and the degree to which contact time or intensity is given to other intervention components.

Feedback of medical images is defined as the individual being shown source images (still or moving) of his or her body (or representations thereof) generated by the procedure in the course of receiving their results. Typically we expect the intervention to consist of the individual being presented with a medical image of his or her body (such as a scan image of an arterial plaque) and explanation being given detailing what the image portrays and the implications this has for their health and behaviour (in this example, outlining the role of smoking in determining arterial health). We will exclude interventions which used library images or images of other people’s scans only (rather than images of or representing the individual themselves) as the focus of risk communication.

Acceptable comparison groups are those that provide:

1. no risk information at all;

2. risk information derived from a non-medical imaging method (e.g. cholesterol test); or

3. personalised health-related risk information derived from medical imaging procedures but presented to the individual without visual feedback.

We will exclude studies that used hypothetical scenarios in which individuals imagined receiving risk information.

***Types of outcome measures***

Primary outcomes

Health-related behaviours that have the potential to modify the risk identified: dietary behaviour; physical activity; smoking; alcohol consumption; attendance for screening; sun protection behaviours; adherence to medication. Eligible trials must have assessed a primary outcome.

Secondary outcomes

- Related risk factors and health-related outcomes including blood pressure, cholesterol level, lung function, BMI/weight, level of atherosclerosis, and disease risk indices such as Framingham Risk Score
- Adverse events and harms, including depression or anxiety.

**Search methods for identification of studies**

***Electronic searches***

The search will update the Cochrane Review [1] searches from 1^st^ September 2009, covering MEDLINE (1946-), Embase (1980-), CINAHL (1982-), PsycINFO (1806- ) and the Cochrane Central Register of Controlled Trials (CENTRAL). We will conduct backwards and forwards citation searches from included studies. Search strategies are provided in Appendix B.

**Data collection and analysis**

***Selection of studies***

Two review authors will pre-screen all search results (titles and abstracts) for possible inclusion, and those selected by either or both will be subject to full-text assessment. The selected articles will be assessed independently by two authors for inclusion, with any discrepancies being resolved by consensus, overseen by a third author acting as arbiter.

***Data extraction and management***

We will extract the following main sets of data from each included study:

• lead author; date;

• study participant inclusion criteria;

• participants (participant diagnoses/condition(s) and demographics: race/ethnicity, gender, socioeconomic status, age);

• study design; randomisation; allocation concealment;

• interventions (content and format of interventions, including details of oral information or description provided; nature of results given to participants; intervention setting and delivery provider; delivery of any co-interventions) and equivalent information on the comparator group(s);

• numbers of participants in each trial arm;

• outcome measures; time(s) at which outcomes were assessed;

• results;

Two authors will independently extract data into a data extraction form, with any disagreements resolved by consensus and a third author acting as arbiter. One author will enter outcome data into Review Manager software, with a second author checking its accuracy. We will contact study authors for additional information about included studies as required.

***Assessment of risk of bias in included studies and quality of evidence***

We will assess and report on the risk of bias in accordance with the RoB 2 tool [3].

Two review authors will independently assess the risk of bias in included studies, with any disagreements resolved by consensus, and with a third author acting as arbiter. RoB 2 addresses five specific domains: (1) bias arising from the randomisation process; (2) bias due to deviations from intended interventions; (3) bias due to missing outcome data; (4) bias in measurement of the outcome; and (5) bias in selection of the reported result. Following guidance given for the tool, we will derive an overall summary ’Risk of bias’ judgement (low; some concerns; high) for each specific outcome, whereby the overall RoB for each study will be determined by the highest RoB level in any of the domains assessed. The summary risk of bias will contribute to a GRADE assessment of the quality of evidence, applied to each primary outcome in terms of the extent of our confidence in the estimates of effects [4]. GRADE criteria for assessing quality of evidence encompass study limitations, inconsistency, imprecision, indirectness, publication bias, and other considerations.

***Measures of treatment effect***

We will separately analyse measures of different primary (e.g. smoking cessation, dietary behaviour) and secondary outcomes. Continuous outcomes for which the precise nature of the measures used differs but the outcomes are comparable, will be integrated and standardised to have common effect sizes, defined as the standardised mean difference (SMD). Where all comparable outcomes use the same measures, mean differences (MD) will be used. The effect size for comparable dichotomous outcomes will be the risk ratio (RR). Where data are presented for more than one time point after the intervention, we will report outcomes for the longest recorded follow-up time. Where outcomes are negatively scored, they will be inverted for integration with positively scored outcomes. For these analyses, we will use final values rather than changes from baseline wherever possible and will contact authors if necessary to obtain these. In any given behavioural domain e.g. smoking, we will use the outcome adjudged to be most important for health in that study context. If multiple indices of a given behavioural outcome are reported, we will use the most stringent and valid measure of behaviour available (e.g. an objective measure such as biochemically validated smoking cessation). Should there be multiple relevant intervention and/or control arms, we will compare those which allow the purest isolation of the intervention effect.

***Dealing with missing data***

We will analyse available data as reported according to participants’ randomised groups. We will not attempt to impute missing data (such as by assuming that participants with missing outcomes were engaging in a risk increasing behaviour e.g. while this practice is common for smoking cessation trials, it cannot be assumed here that even in trials including smoking outcomes, that these will actually concern measures of smoking cessation in smokers).

***Assessment of heterogeneity and publication bias***

We will test for heterogeneity by inspection of overlapping confidence intervals and further quantify this using the I^2^ statistic (which describes the percentage of the variability in effect estimates that is due to heterogeneity rather than sampling error). A value greater than 50% will be considered to represent substantial heterogeneity. Providing there are sufficient data included in our analyses, we will assess for publication bias using funnel plots to informally examine any relationship between study quality and effect size.

***Data synthesis***

We will conduct a narrative synthesis of the included studies, presenting the studies’ key characteristics and results. Providing studies are sufficiently similar in terms of population, interventions and/or outcomes, we will pool the data statistically for each outcome (e.g. type of behaviour). We will obtain pooled effect sizes with 95% confidence intervals using a random effects model - reflecting the heterogeneity likely to arise in the characteristics of included studies – applied on the scale of standardised mean differences and log odds ratios, summarised using forest plots. When different studies report either dichotomous or continuous data for the same outcome, we will combine these data using the generic inverse variance method, and report effect sizes as standardised mean differences. This will follow the methods outlined in the Cochrane Handbook [5] as follows. Standard errors for these studies will be computed by entering the data separately as dichotomous and continuous outcome type data, as appropriate, and converting the confidence intervals for the resulting log odds ratios and standardised mean differences into standard errors. Log odds ratios will then be converted to standardised mean differences by multiplying each by the required constant.

**Changes to protocol from 2010 Cochrane Review [1]**

- The 2010 review analysed separately negatively- and positively-scored but otherwise comparable outcomes. In such instances, we will invert the direction of effect for comparable outcomes and include them in the same analysis.
- We no longer plan to separate clinical and non-clinical populations in our synthesis as we now regard this as an arbitrary and not clearly helpful distinction that in addition will add many more non-essential analyses and reduce the overall clarity of the results.
- When different studies report either dichotomous or continuous data for the same outcome, we plan to combine these data using the generic inverse variance method following methods outlined in the Cochrane Handbook, as opposed to reporting dichotomous and continuous outcomes in separate analyses.
- Single outcome measures within each behavioural domain will be selected if multiple indices of a given behavioural outcome are reported, using the most stringent and valid measure of behaviour available.
- Some of the original secondary outcomes that primarily concern possible psychological mediators/ moderators of any effect of the intervention or that are judged to substantially overlap with more clinically important outcomes will no longer be extracted (e.g. perceived severity and risk of disease, perceived control over the disease risk) to limit the potentially very large number of possible analyses.
- In line with current guidance about the conduct of systematic reviews (e.g. Cochrane MECIR), we will apply a GRADE assessment for each primary outcome.
- Risk of bias for included studies will be assessed using the revised RoB 2 tool [3].

**(Changed or added in October 2021) – Changes to protocol in response to peer review**

- Risk ratios (i.e. relative risks) presented for effect sizes of dichotomous outcomes, instead of odds ratios, having the benefit of being generally more interpretable.
- Only available data used (i.e. there is no longer an assumption of performing risk-increasing behaviour for participants who have dropped out for any outcome).
- Formal exploration of heterogeneity was specified before it was then examined. Heterogeneity was tested using the Chi^2^ test, applying a threshold of p=0.10 due to the likelihood of this being underestimated in small samples, and quantified using the I^2^ statistic, with a value of 50% or greater considered to represent substantial heterogeneity. If the specified thresholds were met for either Chi^2^ or I^2^ values, or both, heterogeneity was further explored. In exploring possible sources of heterogeneity, four key characteristics were considered: i) outcome timepoint, ii) outcome measure; iii) health condition being imaged, and iv) nature of the control group. This was investigated by conducting sensitivity analyses for each of these characteristics where relevant.

**References**

[1] Hollands GJ, Hankins M, Marteau TM (2010). Visual feedback of individuals' medical imaging results for changing health behaviour. *Cochrane Database of Systematic Reviews*; 1:CD007434.

[2] US National Library of Medicine (2020). National Library of Medicine - Subject Headings. https://meshb.nlm.nih.gov/record/ui?ui=D003952 (accessed 12^th^ March 2020).

[3] Sterne JAC, Savović J, Page MJ, Elbers RG, Blencowe NS, Boutron I, Cates CJ, Cheng HY, Corbett MS, Eldridge SM, Emberson JR, Hernán MA, Hopewell S, Hróbjartsson A, Junqueira DR, Jüni P, Kirkham JJ, Lasserson T, Li T, McAleenan A, Reeves BC, Shepperd S, Shrier I, Stewart LA, Tilling K, White IR, Whiting PF, Higgins JPT (2019). RoB 2: a revised tool for assessing risk of bias in randomised trials. *BMJ*; 366:l4898.

[4] Guyatt G, Oxman AD, Akl EA, Kunz R, Vist G, Brozek J, Norris S, Falck-Ytter Y, Glasziou P, DeBeer H, Jaeschke R, Rind D, Meerpohl J, Dahm P, Schünemann HJ (2011). GRADE guidelines: 1. Introduction – GRADE evidence profiles and summary of findings tables. *Journal of Clinical Epidemiology*; 64(4):383–394.

[5] Higgins JPT, Thomas J, Chandler J, Cumpston M, Li T, Page MJ, Welch VA (2019). Cochrane Handbook for Systematic Reviews of Interventions. 2nd Edition. Chichester (UK): John Wiley & Sons.

**Appendix B –**

**Database search strategies**

**MEDLINE (OvidSP - including MEDLINE In-Process, 1946-)**

1. exp diagnostic imaging/

2. diagnosis computer assisted/

3. (mri or magnetic resonance imaging or microscop* or photograph* or holograph* or radiograph* or spectroscop* or stroboscop* or subtraction technique* or thermograph* or tomograph* or transilluminat* or ultrasonograph* or ultrasound or imaging or scan*).tw.

4. 1 or 2 or 3

5. ((show* or presented or presenting or presentation or display* or given or giving or gave or receiv* or provided or providing or provision or view* or expos* or intervention* or motivat* or inform*) adj3 (image* or imaging or picture* or depict* or recording* or scan* or photo or photograph* or radiograph* or tomograph* or thermograph* or holograph* or ultrasound or ultrasonograph* or visual* or their or result*)).tw.

6. (visual* adj3 feedback).tw.

7. 5 or 6

8. 4 and 7

9. (adher* or complian* or noncomplian* or motivat*).tw.

10. patient compliance/

11. health behavior/

12. health knowledge attitudes practice/

13. risk reduction behavior/

14. attitude to health/

15. motivation/ or intention/

16. patient education as topic/

17. counseling/ or directive counseling/

18. or/9-17

19. 8 and 18

20. randomized controlled trial.pt.

21. controlled clinical trial.pt.

22. randomized.ab.

23. placebo.ab.

24. drug therapy.fs.

25. randomly.ab.

26. trial.ab.

27. groups.ab.

28. or/20-27

29. humans.sh.

30. 28 and 29

31. 19 and 30

**Embase** **(OvidSP, 1980-)**

1. exp imaging/

2. exp computer assisted diagnosis/

3. exp radiodiagnosis/

4. exp microscopy/

5. exp photography/

6. exp echography/

7. ultrasound/

8. stroboscopy/

9. thermography/

10. (mri or magnetic resonance imaging or microscop* or photograph* or holograph* or radiograph* or spectroscop* or stroboscop* or subtraction technique* or thermograph* or tomograph* or transilluminat* or ultrasonograph* or ultrasound or imaging or scan*).tw.

11. or/1-10

12. ((show* or presented or presenting or presentation or display* or given or giving or gave or receiv* or provided or providing or provision or view* or expos* or intervention* or motivat* or inform*) adj3 (image* or imaging or picture* or depict* or recording* or scan* or photo or photograph* or radiograph* or tomograph* or thermograph* or holograph* or ultrasound or ultrasonograph* or visual* or their or result*)).tw.

13. (visual* adj3 feedback).tw.

14. 12 or 13

15. 11 and 14

16. (adher* or complian* or noncomplian* or motivat*).tw.

17. patient compliance/

18. exp health behavior/

19. motivation/

20. patient education/

21. counseling/ or directive counseling/ or patient counseling/

22. or/16-21

23. 15 and 22

24. randomized controlled trial/

25. single blind procedure/ or double blind procedure/

26. crossover procedure/

27. random*.tw.

28. placebo*.tw.

29. ((singl* or doubl*) adj (blind* or mask*)).tw.

30. (cross over or crossover or factorial* or latin square).tw.

31. (assign* or allocat* or volunteer*).tw.

32. or/24-31

33. nonhuman/

34. 32 not 33

35. 23 and 34

**Cochrane Central Register of Controlled Trials (CENTRAL)**

#1 MeSH descriptor Diagnostic Imaging explode all trees

#2 MeSH descriptor Diagnosis, Computer-Assisted explode all trees

#3 mri or microscop* or photograph* or holograph* or radiograph* or spectroscop* or stroboscop* or subtraction-technique* or thermograph* or tomograph* or transilluminat* or ultrasonograph* or ultrasound or imaging or scan*

#4 (#1 OR #2 OR #3)

#5 (show* or presented or presenting or presentation or display* or given or giving or gave or receiv* or provided or providing or provision or view* or expos* or intervention* or motivat* or inform*) near/3 (image* or imaging or picture* or depict* or recording* or scan* or photo or photograph* or radiograph* or tomograph* or thermograph* or holograph* or ultrasound or ultrasonograph* or visual* or their or result*)

#6 visual* near/3 feedback

#7 (#5 OR #6)

#8 (#4 AND #7)

#9 adher* or complian* or noncomplian* or motivat*

#10 health-behavior or health-knowledge or risk-reduction or patient-education or counseling or attitude or intention

#11 (#9 OR #10)

#12 (#8 AND #11)

**PsycINFO (OvidSP, 1806-)**

1. exp tomography/

2. exp roentgenography/

3. computer assisted diagnosis/

4. (mri or magnetic resonance imaging or microscop* or photograph* or holograph* or radiograph* or spectroscop* or stroboscop* or subtraction technique* or thermograph* or tomograph* or transilluminat* or ultrasonograph* or ultrasound or imaging or scan*)

5. or/1-4

6. (show* or presented or presenting or presentation or display* or given or giving or gave or receiv* or provided or providing or provision or view* or expos* or intervention* or motivat* or inform*) adj3 (image* or imaging or picture or depict* or recording* or scan* or photo or photograph* or radiograph* or tomograph* or thermograph* or holograph* or ultrasound or ultrasonograph* or visual* or their or result*)

7. exp pictorial stimuli/

8. (visual* adj3 feedback)

9. or/6-8

10. 5 and 9

11. (adher* or complian* or noncomplian* or motivat*)

12. exp compliance/

13. health behavior/

14. health knowledge/

15. risk taking/

16. health attitudes/

17. client attitudes/

18. motivation/ or intention/

19. client education/

20. counseling/

21. or/11-20

22. 10 and 21

23. random*

24. trial*

25. placebo*

26. (singl* or doubl* or trebl* or tripl*) and (blind* or mask*)

27. (cross over or crossover or factorial* or latin square)

28. (assign* or allocat* or volunteer*)

29. treatment effectiveness evaluation/

30. mental health program evaluation/

31. exp experimental design/

32. or/23-31

33. 22 and 32

**CINAHL (1982-)**

S1 MH diagnostic imaging+

S2 MH image processing, computer assisted+

S3 mri or magnetic resonance imaging or microscop* or photograph* or holograph* or radiograph* or spectroscop* or stroboscop* or subtraction technique* or thermograph* or tomograph* or transilluminat* or ultrasonograph* or ultrasound or imaging or scan*

S4 visual* and feedback

S5 S1 or S2 or S3 or S4

S6 adher* or complian* or noncomplian* or motivat*

S7 MH health behavior+

S8 MH attitude to health+

S9 MH attitude to risk

S10 MH intention

S11 behavioral change*

S12 MH patient education+

S13 MH counseling+

S14 MH health knowledge

S15 MH risk taking behavior

S16 MH eating behavior+

S17 MH smoking cessation

S18 S6 or S7 or S8 or S9 or S10 or S11 or S12 or S13 or S14 or S15 or S16 or S17

S19 S5 and S18

S20 randomi?ed controlled trial*

S21 PT Clinical Trial

S22 MH Clinical Trials+

S23 MH Random Assignment

S24 MH Placebos

S25 MH Quantitative Studies

S26 AB (random* or trial or groups or placebo*) or TI (random* or trial or groups or placebo*)

S27 AB (singl* or doubl* or tripl* or trebl*) and AB (blind* or mask*)

S28 TI (singl* or doubl* or tripl* or trebl*) and TI (blind* or mask*)

S29 S20 or S21 or S22 or S23 or S24 or S25 or S26 or S27 or S28

**Appendix C –**

**Outcome selection process**

**Refining the core set of primary and secondary outcomes for analysis**

The outcome set at the point of protocol development was initially informed by that generated by the 2010 Cochrane review. Given the potentially wide range of possible behavioural and secondary outcomes relevant for this review, and a number of new studies to include in the updated review, we could not definitively specify a complete set of outcomes at this stage, but as far as possible primary and secondary outcomes were pre-specified. For example, as specified in the protocol, we excluded those secondary outcomes from the 2010 Cochrane review that primarily concerned possible psychological mediators or moderators of any effect of the intervention (e.g. perceived severity of disease, perceived control over the disease risk). Upon starting the review process, the outcome set was then refined as new outcomes were encountered within studies that appeared to meet our criteria for primary or secondary outcomes, prior to any data extraction or analysis (and thus knowledge of possible results) occurring. This involved determining, in consensus with medically trained team members (JUS,SJG), whether any such outcomes i) met our criteria, and if so ii) should be included or whether outcomes overlapped too greatly (e.g. overlapping with more clinically important outcomes) to justify inclusion given the necessity to reasonably limit the potentially very large number of analyses. Specific examples included whether, first, systolic blood pressure and diastolic blood pressure, and second, BMI and waist circumference, were all necessary to extract – which we judged them to be in both cases. Concerning multiple overlapping measures for a risk marker, specifically for cholesterol measures we prioritised LDL cholesterol, as opposed to multiple possible measures of total cholesterol, high-density lipoprotein (HDL) cholesterol, non-HDL cholesterol, or measures of triglycerides.

Following this process, the final outcome set that the review aimed to extract and analyse comprised:

*Primary outcomes*: smoking, skin self-examination, physical activity, dietary behaviour, medication use, sun protection behaviours, oral hygiene behaviours, tanning booth use, foot care, blood glucose testing.

*Secondary outcomes*: Framingham Risk Score, systolic and diastolic blood pressure, low-density lipoprotein (LDL) cholesterol level, fasting glucose, glycated haemoglobin, body mass index (BMI), waist circumference, skin darkening due to UV exposure, oral health, anxiety, depression, stress.

**Selecting from multiple indices or measures for a given outcome**

As stated in the protocol, if multiple indices of a given outcome were reported, we planned to use the most stringent and valid measure available (e.g. an objective behavioural measure such as biochemically validated smoking cessation). In practice this occurrence was relatively rare, as for 62 of 77 (81%) of comparisons in the review there was only a single outcome measure available per study for that outcome domain (e.g. smoking, healthier diet). However, when this did occur, more detailed criteria were developed for that outcome domain as necessary. Such criteria for specific outcome domains were developed as the outcome set was refined as per the process described above. These criteria – which applied to smoking, dietary behaviour, medication use, sun protection behaviours, oral hygiene behaviours, and skin darkening - are outlined in the table below.

**Specific considerations for outcome domains requiring selection from multiple outcome measures**

| **Outcome domain** | **Notes** |
| --- | --- |
| Smoking | Prioritised measure of smoking cessation/ quitting in (if known/available) participants who were smokers at baseline (as opposed to e.g. frequency of smoking occasions, number of cigarettes smoked). Failing that, prioritised measure of proportion of total participant sample not smoking at follow-up. Then most valid objective measure thereof that was available (i.e. biochemically validated smoking cessation) |
| Dietary behaviour | Prioritised measure relating to most comprehensive set of food products. Prioritised (reduction in) products and nutrients detrimental for health (e.g. consumption of unhealthy foods (as opposed to e.g. of healthy foods)); or consumption of fats and sugars (as opposed to e.g. of fibre or vitamins)) |
| Medication use | Prioritised measure of medication use or adherence by recipient rather than of prescription to recipient. If available, used measure that assessed the degree of adherence/use of new or ongoing medications, prioritising i) focus on statins/cholesterol medications as common in clinical cardiovascular screening contexts, and if not available other cardiovascular health medications; ii) focus on degree of adherence rather than binary use (vs not use) |
| Sun protection behaviours | Prioritised measure that was a composite index of multiple sun protection behaviours. Failing that, prioritised a composite of intentional (vs incidental) sun protection behaviours. Failing that, prioritised a measure of sunscreen use. Valid measures were required to involve actual active sun protection behaviour, so not simply expressed intentions/motivation or preparatory behaviours that are not actively protective (e.g. purchasing sunscreen). Tanning booth use not included and considered as a separate outcome if possible |
| Oral hygiene behaviours | Prioritised measure relating to degree by which behaviour was performed (vs whether a particular binary threshold is reached) i.e. prioritised duration rather than instances. Prioritised composite measure of multiple oral hygiene behaviours, if available, otherwise measure of toothbrushing |
| Skin darkening | Prioritised measure using skin reflectance spectrometry for lightness L* scale readings, using highest part of the human body available for that. NB Consulted with expert researcher in area (author of multiple papers included in review) who confirmed that L* scale (versus b* scale) readings are more sensitive measure of UV exposure |
