# Supplementary material for: Visualising health risks with medical imaging for changing recipients’ health behaviours and risk factors: Systematic review with meta-analysis
Source: PLoS Med. 2022 Mar 3;19(3):e1003920. doi: 10.1371/journal.pmed.1003920 (PMC8893626; doi:10.1371/journal.pmed.1003920)
Supplement: Attachment — Submitted filename: hollands.pdf [file pmed.1003920.s003.pdf]

This paper reports a systematic review with meta–analysis of personalised feedback of medical imaging and health behaviours.

I was asked for a statistical report and I interpret that to include all aspects of the design and conduct of the study.

Points of detail

Page 7 Did the search include articles in any language? There is no apparent mention of language restriction in the search terms in the appendix. If there was a restriction the message I draw from Grégoire et al. (1995); Moher et al. (1996); Egger et al. (1997); Jüni et al. (2002) is that bias is likely although it is hard to predict in advance the extent or direction of it. I would not necessarily commit the authors to translate everything but at least we should know what we are missing.

Page 7 It would be best to give the initials of who did what. Our opinion of the process would be affected if two authors did all the heavy lifting versus independent pairs doing different tasks. The same applies on page 9.

Page 8 If authors did in fact provide extra information then I think they should be acknowledged and the resulting information flagged so we know what we are not going to be able to find in the primary sources.

Page 8 I understand how to synthesise continuous and dichotomous data using, for example, the method described by Chinn (2000) but that is not the inverse variance method claimed here. In fact according to the forest plots inverse variance was used throughout.

Page 8 The authors do not make use of I^2 in their reporting which given the small number of studies in most of the analyses is probably a good thing. Confidence intervals for I^2 are helpful to reveal our uncertainty about the heterogeneity. Even for smoking in Figure 2 with 8 studies the confidence interval of I^2 of 0 to 65% reveals that we essentially have no information here. Those with fewer primary studies are probably worse, I have not checked any others. I know we did not use to use confidence intervals here but I am persuaded by Ioannidis et al. (2007) that we should.

Pages 12 and 13 It seems rather strange to have so many expressed as means. What sort of scale is used to measure medication use for example? I would have expected this to be an essentially binary variable, I either take my tablets or I do not.

Points of more substance

Choosing one outcome

The process for choosing the outcome is not transparent. Who did it and what criteria did they use? Did anybody check? More importantly this wastes information and a multi–level meta–analysis incorporating all outcomes would have avoided this.

Multiple studies from same teams

Several of the forest plots show that the primary studies were done by a small number of teams. This is only to be expected but the results cannot be assumed to be independent which is one of the underlying assumptions of meta–analysis. In this case given the small number of studies I do not think it is worth extending the analysis to incorporate that lack of independence but this is a limitation which needs discussing.

Summary

Requests for more detail and some comments about the analysis strategy.

Michael Dewey

References

S Chinn. A simple method for converting an odds ratio to effect size for use in meta–analysis. *Statistics in Medicine*, 19:3127–3131, 2000.

M Egger, T Zellweger-Zähner, M Schneider, C Junker, C Lengeler, and G Antes. Language bias in randomised controlled trials published in English and German. *Lancet*, 350:326–329, 1997.

G Grégoire, F Derderian, and J Le Lorier. Selecting the language of the publications included in a meta-analysis: is there a tower of Babel bias? *Journal of Clinical Epidemiology*, 48(1):159–163, 1995.

J Ioannidis, N Patsopoulos, and E Evangelou. Uncertainty in heterogeneity estimates in meta–analyses. *British Medical Journal*, 335:914–916, 2007.

P Jüni, F Holenstein, J Sterne, C Bartlett, and M Egger. Direction and impact of language bias in meta–analyses of controlled trials: empirical study. *International Jounal of Epidemiology*, 31:115–123, 2002.

D Moher, P Fortin, A R Jadad, P Jüni, T Klassen, J Le Lorier, A Liberati, K Linde, and A Penna. Completeness of reporting of trials published in languages other than English: implications for conduct and reporting of systematic reviews. *Lancet*, 347:363–366, 1996.
