# Supplementary material for: Visualising health risks with medical imaging for changing recipients’ health behaviours and risk factors: Systematic review with meta-analysis
Source: PLoS Med. 2022 Mar 3;19(3):e1003920. doi: 10.1371/journal.pmed.1003920 (PMC8893626; doi:10.1371/journal.pmed.1003920)
Supplement: Attachment — Submitted filename: PLOSMED REVIEWER RESPONSES October 2021.docx [file pmed.1003920.s004.docx]

**RESPONSE TO EDITORIAL AND REVIEWER COMMENTS**

**Thank you for the opportunity to submit a revised version of our manuscript. We appreciate the detailed and constructive comments from the editors and reviewers and have addressed each of these in turn below. Our responses are reflected in a revised version of the manuscript in which we have highlighted new or substantially altered text resulting from the comments received.**

-----------------------------------------------------------

Requests from the editors:

1. Throughout: Please include line numbers with the revised version.

**RESPONSE: Thank you for highlighting this, which has now been done.**

2. Abstract: Please structure your abstract using the three PLOS Medicine headings (Background, Methods and Findings, Conclusions).

**RESPONSE: This has been done.**

3. Abstract: Please include both 95% CIs and p values for each outcome described in the abstract for the meta-analysis (the p value is misisng for physical activity).

**RESPONSE: This p value for physical activity has now been added. Each outcome is now reported with both 95% CIs and p values.**

4. Abstract: In the last sentence of the Abstract Methods and Findings section, please describe the main limitation(s) of the study's methodology.

**RESPONSE: We have added detail on this to the last sentence of the Methods and Findings section (lines 31-35).**

5. Abstract: Conclusions: Please address the study implications without overreaching what can be concluded from the data; the phrase "In this study, we observed ..." may be useful.

**RESPONSE: In** **line with the suggestion, and comments from other reviewers, we have revised the Conclusions (lines 36-41 and at the end of the Discussion) to both use the suggested wording and also to be more cautious in our interpretation to better reflect uncertainties in the existing evidence. In particular, we now no longer mention any potential benefit for health outcomes as this was not directly assessed in the review, as well as suggesting that further corroboration of these results is needed.**

6. Author Summary: At this stage, we ask that you include a short, non-technical Author Summary of your research to make findings accessible to a wide audience that includes both scientists and non-scientists. The Author Summary should immediately follow the Abstract in your revised manuscript. This text is subject to editorial change and should be distinct from the scientific abstract. Please see our author guidelines for more information: <https://journals.plos.org/plosmedicine/s/revising-your-manuscript#loc-author-summary>

**RESPONSE: We have now included an Author Summary section (lines 51-74).**

7. Methods: Please include all details necessary to understand the methods and analyses, although you note they have been published elsewhere.

**RESPONSE: We have now added substantially more detail about the methods and analyses in the main manuscript in response to helpful reviewer comments. For example, we have now included extensive explanations of the search process (lines 161-171, and Appendix 1 of the protocol which includes the full database searches), how heterogeneity was examined (lines 233-247 as well as throughout the Results section e.g. lines 359-368 and 430-469), the** **GRADE assessment of certainty of the evidence (lines 266-280 as well as throughout the Results section), and a new section in the Results outlining considerations of clinical relevance (lines 498-537). We have included the review protocol as Supporting information as well as an appendix (Appendix 2) that details how outcomes were selected.**

8.Methods: Protocol: Please include the prospective protocol as a supporting information file.

**RESPONSE: This has been done. We have now included the protocol as a supporting information file. We also enclose two substantive appendices to address particular comments from reviewers, namely a full set of electronic database searches (Appendix 1), and a detailed document outlining the process of selecting outcomes for inclusion in the review (Appendix 2).**

9. Methods: Please update the search to the present time (currently December 2019).

**RESPONSE: We have now updated the searches to the end of July 2021, with these details reported in the Abstract (line 12) and Methods (line 161-163). We did not identify any new completed studies eligible for inclusion in the review.**

10. Methods: Please report your SR/MA according to the PRISMA guidelines provided at the EQUATOR site.

<http://www.equator-network.org/reporting-guidelines/prisma/>

Please provide the completed PRISMA checklist.

When completing the checklist, please use section and paragraph numbers, rather than page numbers.

Please add the following statement, or similar, to the Methods: "This study is reported as per the Preferred Reporting Items for Systematic Reviews and Meta-Analyses (PRISMA) guideline (S1 Checklist)."

**RESPONSE: We have now added the requested statement to our Methods section (lines 156-157) and have enclosed a revised version of our PRISMA checklist that now includes section and paragraph numbers alongside page numbers (Supplementary Information). Please note for clarification that we conducted and reported this review in line with the PRISMA 2009 guidelines as that was the current version of the guidelines that were followed when the review was both conducted and submitted (our manuscript was originally submitted on 8^th^ March 2021, with PRISMA 2020 guidelines being published after this, on 29^th^ March 2021).**

11. Results: Please report p values to two decimal places, and three decimal places for p values between .01 and .001. Please use p<.001 for values less than that.

**RESPONSE: This has now been checked and changed throughout the manuscript as necessary.**

12. Discussion: Please present and organize the Discussion as follows: a short, clear summary of the article's findings; what the study adds to existing research and where and why the results may differ from previous research; strengths and limitations of the study; implications and next steps for research, clinical practice, and/or public policy; one-paragraph conclusion. Specifically, please expand the discussion of how your work fits into the context of related and prior literature.

**RESPONSE: We have now revised the Discussion and follow this structure. We have added more detail in several places including in revising the conclusions and describing further important limitations (lines 607-625), and as suggested, have expanded this section particularly in regards to the relationship between this review and previous research and results (lines 558-578).**

13. References: Please use the "Vancouver" style for reference formatting, and see our website for other reference guidelines <https://journals.plos.org/plosmedicine/s/submission-guidelines#loc-references>

**RESPONSE: References have now been formatted in Vancouver style following the submission guidelines.**

14. Figures: Please include both titles and legends for each figure. For example, we suggest indicating in the figure legend for Figures 2, 3, 4, 5, and 6 a brief description of what is shown, and the meaning of the points and whiskers. Please also define any abbreviations used within figures.

**RESPONSE: We have now provided descriptive legends for Figures 2-6, as well as defining the abbreviations used within each Figure (Figure captions on pages 46-48).**

--------------------------------------------------------------------------------------------------------------------------------------

Comments from the reviewers:

Reviewer #1: **Statistical review**

This paper reports a systematic review with meta{analysis of personalised feedback of medical imaging and health behaviours. I was asked for a statistical report and I interpret that to include all aspects of the design and conduct of the study.

Points of detail

1)	Page 7 Did the search include articles in any language? There is no apparent mention of language restriction in the search terms in the appendix. If there was a restriction the message I draw from Gregoire et al. (1995); Moher et al. (1996); Egger et al. (1997); Juni et al. (2002) is that bias is likely although it is hard to predict in advance the extent or direction of it. I would not necessarily commit the authors to translate everything but at least we should know what we are missing.

**RESPONSE:** **The search required an abstract in English but was otherwise not restricted by language. As we have done with previous reviews, we had planned that if articles were identified as potentially eligible based on their title-abstract record, we would then use Google Translate in the first instance to determine potential eligibility of the full-text record, followed by translation if necessary. In practice no such articles were identified. We have now added this clarification to the manuscript (lines 166-169).**

2)	Page 7 It would be best to give the initials of who did what. Our opinion of the process would be affected if two authors did all the heavy lifting versus independent pairs doing different tasks. The same applies on page 9.

**RESPONSE:** **We have now added this detail to the Methods section (lines 210-221 and 261-263) as well as in Appendix 2.**

3)	Page 8 If authors did in fact provide extra information then I think they should be acknowledged and the resulting information flagged so we know what we are not going to be able to find in the primary sources.

**RESPONSE: We have now added text to Table 1 (right-hand column concerning outcomes) to indicate those studies for which additional outcome data was received – see, for example, the entry for Harris (2020). We have acknowledged those study authors who provided the additional data we requested although not by name as we have not asked each of them for their permission to be named (Acknowledgements, lines 706-708).**

4)	Page 8 I understand how to synthesise continuous and dichotomous data using, for example, the method described by Chinn (2000) but that is not the inverse variance method claimed here. In fact according to the forest plots inverse variance was used throughout.

**RESPONSE:** **We appreciate that our previous wording was ambiguous and unclear and so have now clarified our explanation, and checked the revised wording with a senior statistician in our Department who has specialist expertise in meta-analysis (lines 227-232).**

5) Page 8 The authors do not make use of I2 in their reporting which given the small number of studies in most of the analyses is probably a good thing. Confidence intervals for I2 are helpful to reveal our uncertainty about the heterogeneity. Even for smoking in Figure 2 with 8 studies the confidence interval of I2 of 0 to 65% reveals that we essentially have

no information here. Those with fewer primary studies are probably worse, I have not checked any others. I know we did not use to use confidence intervals here but I am persuaded by Ioannidis et al. (2007) that we should.

**RESPONSE: We have now added confidence intervals for I^2^ to our reporting of I^2^ where there were sufficient studies (i.e. at least three) for these to be calculated. The Cochrane software we used for the meta-analysis (Review Manager) does not output confidence intervals for I^2^ so we used the add-on command ‘heterogi’ in Stata for calculating these values. As would be expected and as the reviewer points out, given the relatively few studies in each meta-analysis, confidence intervals are wide. That said, as the reviewer notes we do not place much weight on specific I^2^ values in the review. Reviewer 2 (e.g. Comment 3) and Reviewer 3 (e.g. Comment 8) were in agreement that we should provide more examination of heterogeneity for meta-analyses featuring substantial heterogeneity, something that we had pre-specified in our protocol as indicated by an I^2^ value of ≥50%. We have now presented this (e.g. lines 233-247) and thus used I^2^ and Chi^2^ values to inform these heterogeneity analyses. However, we have also acknowledged that for such relatively small meta-analyses these I^2^ values are accompanied by wide confidence intervals. Indeed, for only one outcome for which we calculated I^2^ confidence intervals and explored heterogeneity did the lower confidence interval not include 0%, namely medication use.**

6)	Pages 12 and 13 It seems rather strange to have so many expressed as means. What sort of scale is used to measure medication use for example? I would have expected this to be an essentially binary variable, I either take my tablets or I do not.

**RESPONSE: Medication use outcomes are commonly presented as continuous data and indeed this is frequently recommended, such as when capturing the degree of adherence or quantity of medication consumed, such as via number of tablets taken in a given timeframe, dosage consumed, percentage of prescribed medication taken, or a score on a questionnaire scale. For example, in this case the Athar (2018) and Rodondi (2012) studies used questionnaire scales to assess the degree of adherence.**

Points of more substance

Choosing one outcome

7)	The process for choosing the outcome is not transparent. Who did it and what criteria did they use? Did anybody check? More importantly this wastes information and a multi{level meta{analysis incorporating all outcomes would have avoided this.

**RESPONSE: We have now provided considerably more detail about this process, particularly in a new appendix (Appendix 2) added to the protocol document. Regarding the possibility of a multi-level analysis, we did not consider there to be a clear need for this given the nature of the existing evidence, and did not propose it in our protocol for this review. We think this is also subsequently justified by the characteristics of the data. There were 77 study comparisons included in the review’s meta-analyses. For most of these comparisons -** **62 of 77 (81%) - there was only one outcome measure available per study for that outcome domain (e.g. smoking, diet) so there was no possibility to include additional outcome measures. This meant that 17 of 23 included meta-analyses used only studies that only had a single possible outcome measure. In the remaining cases where there was more than one outcome measure for a given study, in most instances while more than one alternative outcome measure could in theory have been included in the review, the outcome measures were not similarly relevant and so commonly would be considered inappropriate for inclusion in the review according to our stated criteria. For example, for smoking, there was one study (Rodondi et al, 2012), of eight studies included in the meta-analysis, that reported two eligible outcome measures: point-prevalent abstinence and continuous abstinence. Both are commonly collected in smoking cessation trials, with the former requiring not smoking only in the days before a follow-up (in this case one week), while the latter requires not smoking for the entire period since the quit attempt, in this case 12 months. Not only are these outcomes likely to be substantially correlated and provide little unique information, but continuous abstinence has – by the terms of our protocol – more merit for inclusion, being a more stringent measure with a more proximal link to health benefit and lifelong abstinence. As such, we would not want to include both outcomes in any analysis.**

Multiple studies from same teams

8)	Several of the forest plots show that the primary studies were done by a small number of teams. This is only to be expected but the results cannot be assumed to be independent which is one of the underlying assumptions of meta{analysis. In this case given the small number of studies I do not think it is worth extending the analysis to incorporate that lack of independence but this is a limitation which needs discussing.

**RESPONSE: Thank you for prompting us to comment on this. As the reviewer points out, this issue is most marked for sun protection behaviours, tanning booth use, and oral hygiene behaviours (applying to five of five, two of two, and two of three studies respectively). We have now noted this limitation (Discussion, lines 619-625).**

Summary

Requests for more detail and some comments about the analysis strategy.

**RESPONSE: Thank you for your helpful and detailed comments. Please see our responses above concerning the additional details we have now included in the manuscript.**

--------------------------------------------------------------------------------------------------------------------------------------

Reviewer #2: Ewoud Schuit, Assistant Professor Clinical Epidemiology, Julius Center for Health Sciences and Primary Care, University Medical Center Utrecht

This systematic review and meta-analysis investigated the impact of visualizing health risk based on medical imaging to patients on their health behaviors and risk factors. The systematic review concerns a relevant topic, and the study has been conducted and written down well.

I do have some comments and suggestions to further improve the paper.

Main comments:

1.	I think the current conclusion is formulated too strong. Especially because of the sentence "improve health outcomes". This assumes that healthier behavior results in improved patient outcomes, however, this was not studied in the current review nor was discussed. The paper in general would benefit from linking health behavior and the reduction of risk factor to improved health outcomes in the future, both in the introduction and the discussion of the manuscript. Also, as a reader I would like to know more about how long that change in behavior sticks and whether individuals fall back into their previous 'bad' behavior. Both will have implications for the importance of the outcomes of the current review and meta-analysis.

**RESPONSE: Thank you for these helpful comments. On reflection, we agree with the reviewer and have revised our conclusions to temper them (see also response to the set of editorial comments, Editorial Comment #5 above) in lines 36-41 of the Abstract and 690-694 of the Discussion. We are now more cautious in our interpretation. We no longer mention any potential benefit for health outcomes – as per the sentence mentioned by the reviewer - as this was not directly assessed in the review. In addition, we also emphasise that further corroboration of these results is needed (for example, line 673) and provide substantially more discussion of Limitations (lines 607-625).** **In relation to the more general comments about discussing the relationship between the review outcomes and improved health outcomes, we now** **explicitly make this link in the Introduction (lines 79-85) and discuss this and related issues when we address clinical significance both in the Results (lines 498-537) and Discussion (lines 643-655). Finally, we also now discuss the likelihood that the impacts of these interventions on recipients will endure (Discussion lines 558-566).**

2.	In general, the paper would benefit from additional information on what is considered a minimal clinically relevant difference of the investigated outcomes. Interpretation of SMD's can be difficult, especially when there is no guidance on clinical relevance.

**RESPONSE: We agree with the Reviewer’s comment and have revised the manuscript accordingly. We have addressed this through now presenting illustrative examples in four outcome domains, namely for three primary behavioural outcomes – smoking, healthier diet, oral hygiene behaviours – and a secondary risk marker outcome – systolic blood pressure (these being selected on the basis of the criteria given in the manuscript, lines 500-514). The purpose of this is to help place the results into a more tangible clinical context, as well as to illustrate how similar considerations might be given to other outcomes included in the review.**

**We have not attempted to address this for all of the review’s outcomes, for practical and scientific reasons. In part this is due to the large number and wide range of outcomes that were included in this review, but also that such interpretation requires a degree of expert domain knowledge.** **Where such interpretation is attempted, this inevitably relies on assumptions about the summarised effect, as well as about how that is translated concerning its clinical relevance. Arguably this may require high certainty of results, which is not the case for the results of this review. It may also require clear guidance produced by expert committees or similarly informative evidence in order to judge what constitutes meaningful clinical relevance, made more challenging given the potential for these interventions to be scalable to some degree (i.e. small effect sizes can be important if applied across large populations). Such guidance is rarely available for these kinds of behavioural outcomes. On balance, and in line with these various considerations, we judge that cautiously presenting a small number of illustrative examples, is most appropriate while still having the potential to be informative to readers. This also reflects that in this review we have generally focused more attention on considering the broad set of findings holistically to inform broad intervention approaches, which was always the aim, rather than seeking to generate definitive evidence and related clinical guidance for any specific intervention context or behavioural domain.**

**To address this, we added a new section to Results (lines 498-537), with the subheading ‘Assessing clinical relevance of effect estimates’. We considered placing this new text in the Discussion but on balance think it is better placed in Results due to it including a range of numeric data and because it follows on directly from the meta-analysis results earlier in the same section of the paper. To complement this more detailed section in the Results, we have also added some text on this topic to the Discussion section (lines 644-656).**

3.	Statistical heterogeneity was assessed based on the chi-square test and i2. I think it would be helpful to further investigate heterogeneity for the outcomes with high heterogeneity. So, can it be explained by certain study characteristics? Did the authors consider for example meta-regression or subgroup analyses to investigate reasons of heterogeneity?

**RESPONSE: We did not anticipate at the outset that there would be sufficient studies within meta-analyses to allow appropriate use of meta-regression or subgroup analyses – the Cochrane Handbook advises that there should be at least ten observations available for each characteristic (**[**https://training.cochrane.org/handbook/current/chapter-10#section-10-11**](https://training.cochrane.org/handbook/current/chapter-10#section-10-11)**). This was the case, with no single meta-analysis including more than eight studies. However, we agree with the reviewer (as well as the other reviewers who made similar suggestions) that investigating meta-analyses featuring substantial heterogeneity was merited. To do so, we conducted a series of sensitivity analyses to assess the impact of a set of four key characteristics that varied between studies in the review and that we judged to have the potential to be key sources of heterogeneity. While we did not specify these characteristics at the protocol stage, meaning these analyses were somewhat exploratory in nature, these characteristics were identified prior to any analysis and so without awareness of the likely results of this.** **To describe the process we used, we have now added text to the Methods (lines 233-247). We have then added the findings to the Results section following presentation of the relevant primary outcomes (between lines 319-407) and secondary outcomes (lines 430-469). We have also added the fact that we were only able to interrogate heterogeneity to a limited degree as a key limitation of the review (Discussion, lines 614-619).**

Minor comments:

Abstract

1.	The literature search is close to one year ago. An update or at least an assessment of the literature published since 15th July 2020 would be recommended.

**RESPONSE: We agree that the searches required updating and have now updated the searches to 28^th^ July 2021, as was also advised in the editorial comments we received (Abstract (line 12) and Methods (lines 161-163)). We did not identify any new completed studies eligible for inclusion in the review.**

2.	I would be OK with leaving p-values out of the abstract and only presenting 95% CIs.

**RESPONSE: We have been advised in the editorial comments (see editorial comment 3 above) to include p-values in the Abstract and so have followed the editorial request.**

3.	the authors indicate in the abstract that for "other behavioral outcomes...point estimates favored the intervention but were not statistically significant". Can the authors elaborate on clinical relevance, despite these effects not being statistically significant?

**RESPONSE: We have responded above with a fuller response to this reviewer’s helpful main comment about assessing clinical relevance above (Reviewer 2, main comment 2). As we have explained, and set out in the manuscript (lines 498-537) we purposefully assessed this only for a subset of primary outcomes, specifically those for which a meta-analysis of multiple studies indicated an at least small and statistically significant effect – smoking, healthier diet, and oral hygiene behaviours (as well as one secondary outcome). For these outcomes we sought to interpret the observed effect sizes in terms of the potential to represent a clinically significant or meaningful impact. We judged it overly speculative given the intended scope of this review to attempt to ascribe clinical significance to other behavioural outcomes for which analyses were not clearly statistically significant.**

Methods

4.	Search. I know some details can be found in the first Cochrane publication, but the current manuscript would benefit from some additional details, e.g.:

a.	Did the authors consider searching for ongoing trials, e.g., clinicaltrials.gov? If any trials are ongoing, this may be useful to know and potentially mention in the Discussion section.

b.	Did the authors search references of identified studies?

c.	Did the authors search grey literature?

d.	Were there language restrictions?

**RESPONSE: Regarding point (a), we searched the Cochrane Central Register of Controlled Trials (CENTRAL) which includes trial registration records from two major registries: NIH’s** **ClinicalTrials.gov (the largest clinical trials registry), and the World Health Organization’s International Clinical Trials Registry Platform (ICTRP). As such, our search strategy had the potential to capture ongoing trials, at least for trials that had been registered in these locations. In conducting the search process, we identified three ongoing studies which appear to meet our eligibility criteria and so would likely be included in any future update of this systematic review. We have now mentioned these studies in the Discussion section as suggested (lines 679-681).**

**For point (b), yes, we conducted forwards and backwards citation searches. This is reported in the Methods section (line 162-163).**

**For c, grey literature was searched in two ways. First, as mentioned in our response above to point (a), CENTRAL includes records from two predominant clinical trial registries being ClinicalTrials.gov, and the International Clinical Trials Registry Platform (ICTRP). Second, the Embase searches, as well as the backwards and forwards citation searching using Google Scholar, encompass grey literature including conference proceedings and preprints. These details have now been made explicit in the manuscript (lines 163-166). Beyond this coverage, we did not search any additional electronic databases that are exclusively limited to grey literature sources.**

**For point (d), as mentioned in our response to the first comment from the statistical reviewer (Reviewer 1, Comment 1), the search was not restricted by language for ultimate inclusion in the review, but was for abstract assessment. This has now been clarified in the manuscript (lines 166-169).**

5.	Appendix 1 presents the detailed MEDLINE search strategy. Please also provide search strategies for the other electronic databases. And in addition to that, provide the number of identified records for each electronic database in separate boxes on the first row of Figure 1.

**RESPONSE: This has now been done with all search strategies included in Appendix 1 of the protocol document, and the yields per database included in the PRISMA diagram (Figure 1).**

6.	Please explain why nonrandomized trials were not considered.

**RESPONSE: We excluded non-randomised studies because, first, compared with RCTs, non-randomised studies rely on more stringent and sometimes non-verifiable assumptions in order to confer confidence that the risk of systematic differences between comparison groups beyond the intervention of interest (i.e. confounding) is sufficiently low to permit valid inferences about causal effects. Second, as randomised controlled trials are practicable and possible to conduct within this intervention context, and have been conducted in sufficient numbers to merit a systematic review - evidenced by the preceding 2010 Cochrane review and our knowledge that additional trials had been conducted since - we judged it appropriate to limit our focus to these designs that are most appropriate for assessing the effect of these interventions.**

7.	the control group in identified studies either encompasses personal risk information or no such information. Did results of the intervention differ across these control groups, e.g., were seen effects stronger in the latter group of studies?

**We did not plan this specific analysis and given the considerable number of possible analyses and with relatively few studies within each meta-analysis, when considering the impact of heterogenous control/comparison groups we focused instead on what we consider an overlapping but more directly relevant (for this specific review) factor of whether control participants also underwent the medical imaging procedure, but without receiving visual feedback. For those analyses in which heterogeneity was observed and which were heterogenous in their control groups in this regard - namely medication use, Framingham Risk Score, diastolic blood pressure, LDL cholesterol, glycated haemoglobin, and waist circumference - as reported in the Results we did not find that removing studies that differed meaningfully altered results or interpretation. That said, we agree with the reviewer that the overlapping but different aspect they mention is potentially of interest. This is something we would hope that a future version of this review could meaningfully address with a larger number of studies for any given meta-analysis allowing meta-regression or similar to be conducted. This could systematically disentangle intervention and comparison components to estimate their relative importance in modifying the observed effects. This is reflected in comments made in the Limitations section (lines 614-619).**

8.	in the data extraction and risk of bias assessment paragraph, consider providing initials of those that performed the screening and extraction.

**RESPONSE: We have now added this detail to the Methods section (lines 210-221 and 261-263) as well as in Appendix 2.**

9.	effect sizes for dichotomous outcomes were presented as odds ratios. I was wondering why risk ratios were not considered, provided that included studies were prospective RCTs, meaning absolute risks could be estimated. Risk ratios are easier to interpret than odds ratios.

**RESPONSE: We agree that risk ratios (i.e. relative risks) have the benefit of being more interpretable to most people and so now present risk ratios when expressing effect sizes of dichotomous outcomes (e.g. for the smoking outcome, line 325), as opposed to the odds ratios that were previously included in the manuscript.**

10.	Did the authors consider to study publication bias? If not, please provide an explanation in the manuscript.

**RESPONSE: We did not formally examine publication bias due to the small numbers of studies for each meta-analysis, with Cochrane Handbook guidance advising at least 10 studies are needed for meaningful and reliable appraisal of this for any given meta-analysis through formal testing. However, we sought to minimise the risk of publication bias by including grey literature (see our response to Reviewer 2, Comment 4c, where this is described). This is now mentioned as a limitation of the review (Strengths and limitations of this review, lines 610-612).**

Results

11.	for medication use, tanning booth use, waist circumference, and FRS, the i2 is high (>70%), which makes it questionable whether data should be pooled in the first place. Did the authors examine where this heterogeneity originated from or tried to account for this (e.g., using meta-regression)?

**RESPONSE: We appreciate that philosophies vary as to when meta-analysis becomes inappropriate or not. Our view is that a degree of heterogeneity is highly likely to be observed in the context of behavioural interventions - although not inevitable, and several of our analyses were not appreciably heterogenous (albeit noting wide confidence intervals around values). We think it is therefore preferable to conduct and present all analyses in a consistent way for all outcomes rather than impose cut-offs for no longer conducting meta-analyses with a given level of heterogeneity (not least because these are essentially arbitrary and it is likely – as the statistical reviewer highlights – that heterogeneity estimates for small meta-analyses are usually highly uncertain). In response to this comment and that of Reviewer 2, we now report explorations of heterogeneity (see our response to point 3 from reviewer 2 for details) that while limited, provide some reassurance that this does not meaningfully impact results or interpretation.**

**More generally, meta-analysis has known advantages and disadvantages. One particular important advantage in our view is that meta-analysis enables a relatively consistent and transparent way of integrating and analysing data, providing heterogeneity is explored and is not consistent and extreme, and that limitations are appropriately acknowledged. Throughout the manuscript we have now aimed to be more explicit and comprehensive about the limitations of our analyses and the caution that should be applied to the results we present.**

12.	sun protection. Pooled analysis "showed no significant effect". Is and SMD of 0.14 indeed considered "no significant effect", both clinically and statistically? Please change to "statistically significant" if that is what is meant here. A similar comment can be made about blood glucose testing.

**RESPONSE: The 95% confidence intervals (which we checked in both the manuscript and Figures) for these analyses in both cases overlap 0 and so we intended to point out that these are not statistically significant by the imposed significance value of 5%. As such, as the reviewer suggests, we have made this clear for sun protection by changing the text to “no statistically significant effect” (line 373-374). For blood glucose testing, we have used the term “no clear evidence” (line 403) to reflect that while the point estimate suggests a small effect, the confidence intervals and p-value mean that this cannot be accepted as a true effect. In general, in terms of interpreting the meta-analytic effects as they relate to clinical significance, please see response to Reviewer 2, Comment 2 in which we describe how we have now addressed this in the revised manuscript.**

13.	consider adding "certainty of evidence" to the results section where results for each of the primary and secondary outcomes are presented. An explanation on GRADE and when the authors considered to downgrade could be added to the methods section.

**RESPONSE: As suggested, in the Methods section, we have now included a more general explanation of the GRADE approach (lines 266-280). We have also included additional GRADE information into the Results section, namely adding brief text on certainty of evidence for each primary outcome, as well as describing any decisions to downgrade certainty in the ‘Assessment of risk of bias and certainty of evidence’ in more detail than we had previously (lines 473-496).**

Discussion

14.	There are several additional topics that could be addressed in the discussion section:

a.	As indicated earlier, the paper would benefit from linking evidence on health behavior change to change in health outcomes. Also, to know more about how long that change in behavior sticks and whether individuals fall back into their previous 'bad' behavior. Both will have implications for the importance of the outcomes of the current review and meta-analysis.

b.	Interventions were generally an hour or less and took place once. How big of an effect would you expect from such an intervention? Could be something to elaborate on a little bit in the discussion.

c.	I would be interested in reading more about the implications of the risk of bias and identified heterogeneity across certain outcomes.

**RESPONSE: Concerning a) please see our previous response on this topic to the earlier comment #1 from the same reviewer. Essentially, we agree and have made changes to reflect this. We now explicitly make the link between the review outcomes and health outcomes in the Introduction (lines 79-85) and discuss this and related issues when we address clinical significance both in the Results (lines 498-537) and Discussion (lines 643-655). We also now discuss the likelihood that the impacts of these interventions on recipients will be sustained (Discussion lines 558-566).**

**Concerning b) we agree that large effects would not be expected. We comment on this and how in this sense the results appear to align with other comparable interventions (lines 633-641). Relatedly, we have also now made several changes to attempt to place the effect sizes in context as regards their clinical relevance (see, for example, our response to Reviewer 2, Comment 2).**

**c) As per our responses to several other reviewer comments we have now provided considerably more information and discussion around heterogeneity (e.g. please see our response to Reviewer 2, Comment 3) as well as concerning limitations of studies and the syntheses thereof, including risk of bias (for example, see our response to the above comment (Reviewer 2, Comment 13).**

--------------------------------------------------------------------------------------------------------------------------------------

Reviewer #3: Thank you for the opportunity to review this manuscript. It is as well-written and important piece of work. I have read the manuscript with great interest and have a few comments for the authors to reflect upon.

1.	P7 1st para last sentence: Did the author exclude studies referred to on the last sentence which commenced with "Studied assessed the intervention effect relative to a comparison group that was either provided…"? The sentence is incomplete.

**RESPONSE:** **This section has now been rewritten to aim to improve its clarity (lines 194-200).**

2.	P7 2nd para on primary outcome: "Primary outcomes were health-related behaviours with the potential to modify communicated health risks". The phrase "to modify communicated health risks" is confusing. Could this be rephrased?

**RESPONSE: We have now rephrased this to avoid the difficult wording (lines 201-202).**

3.	P7 2nd para on secondary outcome: Did the author focus on adverse events and harms, such as anxiety, depression or stress which are related to the intervention only? Did the included studies measure the intervention-related events or harm, or more in general?

**RESPONSE: Beyond unwanted adverse effects on health behaviours and risk factors, which were routinely identified in the review process, in terms of harms, we focused on gathering any reported data for anxiety, depression, or stress outcomes, but also sought any mention of any more general harms. No such harms were reported (lines 427-429).**

4.	P8 2nd para: Please clarify the following statement as the use of "combined these data" can lead to misunderstanding. Didn't the author transformed dichotomous data using inverse variance method, so they could finally report the effect sizes as standardised mean differences for all studies homogenously?

"When different studies reported dichotomous and continuous data for the same outcome, we combined these data using the generic inverse variance method."

**RESPONSE: As per our response to Reviewer 1, Comment 4, we appreciate that our previous wording was ambiguous and unclear and so have now clarified our explanation, and checked the revised wording with a senior statistician in our Department who has specialist expertise in meta-analysis (lines 227-232).**

5.	P8 3rd para: The approach the authors used to select the outcome variables (the one with the longest follow-up time, the one which was judged to be most important for risk reduction, and the one which was most stringently measured) lead to heterogeneity of the outcomes measured in different studies. For example, it is inappropriate to use the data points from the longest available follow-up as the outcome would reflect intervention effects at different time points after the intervention. And hence, it is misleading to combine their effects in a meta-analysis. Shouldn't the most common follow-up time, for example, be used and results from all follow-up time are reported

separately?

**RESPONSE: Using the longest follow-up time is a common approach, albeit one with important limitations as with any approach, and is listed as one viable option for analysis in the Cochrane handbook (**[**https://training.cochrane.org/handbook/current/chapter-06#section-6-2-4**](https://training.cochrane.org/handbook/current/chapter-06#section-6-2-4)**.), which also mentions the same key limitation as raised by the reviewer – in that it may induce heterogeneity. A notable benefit, in our view, is that it offers a practical means of including as many eligible studies as possible in a relatively concise and comprehensible set of analyses. The alternative approach suggested by the reviewer has likely benefits of lower heterogeneity but has drawbacks too, in for example, being more likely to produce a complex and unwieldy set of analyses that are difficult to interpret as a whole. Given the number of included studies, these could have multiple analyses for each outcome domain, each comprising very small numbers of studies. In relation to this specific review, we also think the nature of the available data justifies our decision. First, of 77 comparisons included in meta-analyses, 60 (78%) had only one reported timepoint so there was limited opportunity to select from outcome timepoints to maximise their commonality. Second, and relating to the first point, because the actual range of different timepoints used by studies was relatively narrow –12 month follow-up periods were most common – this meant that what were the longest timepoints available within studies were usually relatively concordant with the modal timepoint. Third, we have now detailed in the manuscript how we have addressed heterogeneity, and in analyses where there was marked heterogeneity, one of the factors we explored was heterogeneity of outcome timepoints. As reported, the sensitivity analyses we conducted to remove studies that differed from the modal timepoint, did not meaningfully alter results or interpretation.**

6.	P8-P9 on missing data: One can also challenge the approach the authors used to handle missing data. Wouldn't the approach of "assuming that participants with missing outcomes were engaging in the risk increasing behaviour" bias the estimates?

**RESPONSE: While this approach to missing binary data is standard in smoking cessation trials (e.g. ‘Russell standard’ approach) the reviewer’s comment has led us to decide to no longer apply it to the smoking meta-analysis because not all the trials that assessed smoking behaviour were trials of smokers (as is the case in smoking cessation trials, and where this assumption is considered reasonable). We also agree with the reviewer more generally as it applies to other outcomes, particularly given these assumptions are less widely made for other behaviours than they are for smoking. As such,** **we have used the available data (i.e. no longer making an assumption of risk-increasing behaviour for participants who have dropped out). This only minimally altered meta-analytic results for smoking, physical activity, healthier diet, skin self-examination, and medication use; point estimates for these effects were entirely unchanged for smoking, physical activity, healthier diet, and medication use. The effect size estimate for skin self-examination did change, albeit slightly (from RR of 2.89(p=.001) to RR of 2.71(p=.002). In summary, changing our analyses in line with the reviewer’s comment, to include only available data did not meaningfully change any results or their interpretation.**

7.	P13 on secondary outcome: If possible, add brief detail of the follow-up, especially for the mental health outcomes as the negative effect of intervention on mental health might decay over time.

**RESPONSE: We have now added details of the outcome timepoints for secondary outcomes in the Results section (lines 411-428) as well as included this detail for each study in Table 1 (right-hand column listing primary and secondary outcomes).**

8.	Results: Some of the secondary outcomes, such as Framingham Risk Score, waist circumference and oral health were significant and had I2 ≥50%. Should the authors consider doing subgroup analysis to explain the high heterogeneity? I would suggest more discussions about the heterogeneity observed in this study.

**RESPONSE: As per our response to Reviewer 2, Comment 3, we have now provided additional detail describing examination of heterogeneity. This included exploring this in multiple outcomes including those highlighted by the Reviewer i.e. Framingham Risk Score, waist circumference, and oral health. We have added text to the Methods (lines 233-247) to describe the process we used, and included the findings thereof in the Results section following presentation of the relevant primary and secondary outcomes.**

9.	Results: Many of the studies included have very small sample size. At least the authors can comment on this in the Discussion section.

**RESPONSE: We now comment on this in the Discussion where we present some key limitations of the review (e.g. lines 609-610). Relatedly, when reporting certainty of the evidence we also now describe more fully why meta-analyses were downgraded due to small samples which in most cases involved fewer than 500 participants (lines 491-493).**

10.	Conclusion: I think one of the benefits of the widespread and becoming less expensive medical imaging technologies such as carotid ultrasound is its potential to outreach wider population. These technologies could be used for screening of large portion of the population with moderate risk but asymptomatic diseases, serving the purpose of primary prevention for non-communicable diseases.

**RESPONSE: We have added text to the Discussion to reflect this, in that we agree that as the technologies become cheaper and more available, a wider primary prevention role could become more justifiable, albeit subject to evidence of benefit (lines 68-671).**

Reviewer #4:

In their systematic review and metanalysis (SR-MA) of visualising health risk with medical imaging the authors show risk-reducing behaviours and reducing risk factors.

The reviewer has the following comments/questions on the submit:

1)	The authors are encouraged to formulate a PICO(T) for the SR-MA and especially motivate the great variety of outcome variables.

**RESPONSE: We have now restructured the section of the methods that contained our inclusion and exclusion criteria using PICO subheadings in order to make the PICO components clearer (lines 174-208).**

2)	Primary and secondary outcomes: The authors should motivate the choice of health risks (primary outcome mandatory for including studies) and secondary risk factors. It appears that for example Framingham Risk Score is more likely to reflect future hard endpoints than for example oral hygiene behaviours.

**RESPONSE: First, we agree that the relationship is generally closer – and evidence stronger – between the secondary risk marker outcomes and hard clinical endpoints, than it is for the primary behavioural outcomes. However, in our view this does not negate that a consistent and important challenge in medicine and public health is how to change behaviour to reduce health risk. Furthering this understanding is important for several reasons. First, behaviour is commonly the entity that is being targeted by these kinds of interventions as a proximal mechanism by which risk reduction is achieved (measured in subsequent risk marker changes), and so it is logical for behaviour to be the focus when assessing these interventions. Second, more generally, understanding effects of healthcare interventions on behaviour is key because such approaches are able to complement those biomedical approaches to healthcare that – in relative terms at least - bypass cognition and behaviour and impact clinical endpoints more directly. Examples include surgical interventions or drug treatments (although of course the latter often rely on adherence behaviours). Finally, and relatedly, a primary focus on behaviour is justified because developing understanding of how health communication intervention may work could inform the development of other new or complementary strategies for health communication.**

3)	The title of the previous Cochrane report "Visual feedback of individuals' medical imaging results for changing health behaviour" appears more distinct and clear compared with the title in the manuscript.

**RESPONSE: Thank you for this helpful feedback. We agree that the title could be clearer although we have previously received feedback on the Cochrane title that the term ‘Visual feedback’ was unclear and that ‘medical imaging’ would be better understood. As such, we have now attempted to integrate and simplify these options and propose to use the following title: “Visualising health risks with medical imaging for changing recipients’ health behaviours and risk factors: systematic review with meta-analysis”.**

4)	Abstract; objective: The meaning of "To assess the impact on risk-reducing health behaviours and risk factors of communicating to individuals images representing their bodies derived from medical imaging procedures." is somewhat unclear and should be rephrased.

**RESPONSE: We have rephrased this as “…to assess the impact on behaviours and risk factors of feeding back to individuals images generated via medical imaging technologies in assessing their current disease status or risk” (lines 6-8).**

5)	Methods, page 7 1st para last sentence: "Studies assessed the intervention effect

relative to a comparison group that was either provided with personalised risk information derived from a non-medical imaging method (e.g. cholesterol test) or from an imaging method but without any visual feedback, or that provided no personalised risk information (e.g. provided only generic health risk information)." Do you mean that these studies were also excluded; please clarify.

**RESPONSE: We have now entirely reworded this section to make it clearer (lines 194-200), under a new Comparison subheadings as per our response to Comment 1 from this reviewer.**

6)	Skin darkening is listed in the results of secondary outcome but is not mentioned in the Methods section.

**RESPONSE: This has now been added (Inclusion and exclusion criteria, line 207).**

7)	Methods, page 8, para 3: The authors state that "If multiple indices of a given behavioural outcome were reported, we used the most stringent and valid measure available (e.g., an objective measure such as biochemically validated smoking cessation)." The authors should describe this in more detail and give more examples.

**RESPONSE: We agree with the reviewer and comments from other reviewers making a similar point and have now provided considerably more detail concerning how outcomes were selected, in particular an Appendix (Appendix 2) describing the process of outcome selection.**

8)	Results; table 1: The authors do not consequently specify different kinds of plaque (dental plaques, carotid atherosclerotic plaques); this should be clarified.

**RESPONSE: Thank you for this observation. We have now clarified this in all instances where the term ‘plaque’ is used, both in Table 1 and in the main manuscript text.**

9)	Results, table 1 in the column primary outcome selected for review needs to be revised in more detail. For example:

Ref 36 Systolic blood pressure, cholesterol (total, TG, LDL), fiber intake, BMI reported in publication but is not described in Table 1 in SR-MA; decrease in fat intake is not an established factor of improved health.

Ref 38 FRS, cholesterol (total, LDL), blood pressure (syst/diast), medication reported in publication but is missing in the Table 1 in manuscript.

Ref 40: Electron beam tomography (CT) of what?? FRS (primary endpoint in publication), blood pressure, LDL and more reported in publication but not included in Table 1 in manuscript.

Ref 42 FRS reported in publication but not in Table 1 in the manus

Ref 43 FRS reported in publication but not in Table 1 in the manus

**RESPONSE: First, as suggested by the Reviewer, we have now added the secondary outcomes that we included in our analysis into Table 1. We had previously limited this to just primary outcomes to ensure the Table was concise, but agree with the reviewer that completeness is more important here. Relatedly, we have also now checked there are no missing outcomes i.e. where those were included in our analysis but not listed in the Table, and clarified the nature of the medical imaging for the O’Malley (2003) study (this was Reference 40 referred to in the above comment).**

**Second, for what was Reference 36, the Lederman (2007) study, we have continued to include data for decrease in fat intake as a relevant primary outcome – this being selected as the outcome most consistent with our protocol (considering “the potential to modify the risk identified”) and criteria specific to diet-related outcomes. We do not agree with the suggestion that “decrease in fat intake is not an established factor of improved health”. Overall fat intake is an indicative outcome of increased risk, largely given that it captures consumption of different types of fats, including those known to be more or less risk-increasing - even if in an ideal world a more detailed measurement capturing independently those different fat types would be preferable. We consider this to be a credible position, both in the present day but also for 2007 when this study was conducted. For example, 2001 NCEP expert panel clinical guidelines** *(doi.org/10.1001/jama.285.19.2486)* **referenced by Lederman (2007), and which they state informed the content of the feedback given in the study, emphasise that limiting total fat intake to “25-35% of total calories” was regarded as beneficial to health. Other core guidelines from that point in history also reflect this including the 2000 Dietary Guidelines** *(https://health.gov/our-work/nutrition-physical-activity/dietary-guidelines/previous-dietary-guidelines/2000)* **and 2005 Dietary guidelines** *(https://health.gov/our-work/nutrition-physical-activity/dietary-guidelines/previous-dietary-guidelines/2005)* **which suggest limiting total fat intake and keeping this to between 20 to 35 percent of calories. More generally, it appears that until at least 2015 the emphasis in changing dietary fat consumption was on total fat and it was only then that guidelines moved away from aiming to limit such intake** (*Mozaffarian and Ludwig (2015): doi.org/10.1001/jama.2015.5941; Wang and Hu (2017): doi.org/10.1146/annurev-nutr-071816-064614)***. It is reasonable to assume that this was reflected in what was communicated to patients and why total fat intake was considered to be a risk-reducing health behaviour by the study.**

11)	In the discussion, the relationship between the effect of change in risk/risk factors and health outcomes (hard endpoints such as cancer, CVD, premature death) should be brought up in more detail.

**RESPONSE: As per our response to Reviewer 2 (Main comment 1) amongst others, we now explicitly make this link in the Introduction (lines 79-85) and discuss this and related issues when we address clinical significance both in the Results (lines 498-537) and Discussion (lines 643-655), including the specific example of a key risk factor, systolic blood pressure (lines 532-537).**
